# The Role of Anterior Segment Optical Coherence Tomography in Glaucoma

**DOI:** 10.1155/2012/476801

**Published:** 2012-08-01

**Authors:** Sarwat Salim

**Affiliations:** Hamilton Eye Institute, University of Tennessee, 930 Madison Avenue, Suite 470, Memphis, TN 38163, USA

## Abstract

The anterior segment optical coherence tomography provides an objective method to assess the anterior segment of the eye, including the anatomy of the anterior chamber angle. This technology allows both qualitative and quantitative analyses of the angle and has shown potential in detecting and managing angle-closure glaucoma. In addition, it has a role in identifying pathology in some forms of secondary open-angle glaucoma and postsurgical management of glaucoma. Limitations of this technology include its cost and inability to visualize well structures posterior to the iris, such as the ciliary body. This paper focuses on potential benefits and limitations of anterior segment optical coherence tomography when compared with conventional gonioscopy and ultrasound biomicroscopy. Various clinical entities will be described to discuss its potential role in glaucoma practice.

## 1. Introduction

Glaucoma is a progressive optic neuropathy characterized by structural changes in the optic nerve head with corresponding changes in the visual field. While the final pathway involving structural and functional loss is similar for various types of glaucoma, a comprehensive evaluation of the drainage angle is critical for accurate diagnosis and appropriate therapeutic intervention. In the United States, the most common type of glaucoma is primary open-angle glaucoma, whereas primary angle-closure glaucoma is the major form in other parts of the world [1–4]. There is a wide spectrum of anatomical variation in the drainage angle in normal and affected eyes. Many patients may present with narrow or occludable angles without any other abnormality; some may have primary angle closure with peripheral anterior synechiae and/or elevated intraocular pressure; yet others may have primary angle-closure glaucoma with optic nerve damage. Furthermore, forces at different anatomic levels in the eye may be responsible for the pathogenesis of angle closure: the iris (pupillary block), the ciliary body (plateau iris), the lens (phacomorphic glaucoma), and posterior to the lens (malignant glaucoma) [5]. Therefore, assessing anterior chamber angle anatomy and surrounding structures with anterior segment imaging is of tremendous importance for identifying individuals in the early stages of the disease and guiding therapeutic decisions. 

## 2. Discussion

### 2.1. Gonioscopy

Gonioscopy remains the reference standard for assessing anterior chamber angle in the eye. It is inexpensive, rapidly performed at the slit-lamp, permits dynamic visualization of the entire angle quadrant, and allows indentation differentiating between appositional and synechial angle closure. However, this is a subjective technique and is easily affected by patient cooperation, examiner's skill, type of lens used, direction of gaze, inadvertent pressure on the cornea, and environmental illumination [6]. Furthermore, it does not provide quantitative evaluation of the angle and is also limited in visualizing structures posterior to the iris. Different classification systems may cause variability in angle assessment [7, 8]. Interobserver variability is reported to be only moderate in some studies [3, 9, 10]. 

Imaging of the anterior segment of the eye offers an objective method for visualizing the angle and adjacent anatomical structures. In addition to qualitative analyses, some imaging modalities permit quantitative analyses that can be used to monitor change or progression over time. Several imaging devices are described below to understand potential benefits and limitations of anterior segment optical coherence tomography (AS-OCT).

### 2.2. Ultrasound Biomicroscopy (UBM)

UBM uses high-frequency ultrasound (35–100 MHz) to provide high-resolution images of the angle. A typical UBM system (Paradigm Medical Industries, Salt Lake City, UT) uses a 50-MHz transducer and provides an axial resolution of 25 um, lateral resolution of 50 um, tissue penetration of 5 um, and 256 A scans at a rate of eight frames per second [11]. This earlier model is limited in its ability to provide images in only one quadrant of the eye at a time. However, the newer units, such as OTI (Ophthalmic Technologies, Toronto, Canada) and VuMax II (Sonomed, Inc., Lake Success, NY, USA) acquire images of 180° of the eye in one frame. Recently, even higher-frequency ultrasound devices (Iscience, Mountain View, CA) permit visualization of Schlemm's canal and trabecular meshwork by using an 80-MHz probe. However, using higher frequencies compromises the quality of images posterior to the iris. UBM has shown good agreement with gonioscopy in assessing the anterior chamber angle [12, 13]. Although UBM is a useful technology capable of providing both qualitative and quantitative analyses, its major advantage lies in its ability to visualize structures posterior to the iris to detect various causes of secondary angle closure, such as plateau iris, ciliary effusions, or iridociliary masses [11, 14]. The disadvantages of UBM include required supine position, use of anesthesia, need for a skilled examiner, longer image acquisition time, and contact with the eye using a cup with a coupling medium or a probe that can lead to corneal abrasion or potential infection. 

### 2.3. Scheimpflug Photography

The Pentcam (Oculus, Lynnwood, WA) uses a rotating Scheimpflug camera to provide a 3-dimensional image of the anterior segment of the eye [15, 16]. Although this noncontact device allows rapid image acquisition and provides measurements of anterior chamber depth and volume, corneal thickness, and lens thickness, it does not provide detailed information of the angle recess because of light-scattering and has limited application in documenting angle closure.

### 2.4. EyeCam

The EyeCam (Clarity Medical Systems, Pleasanton, CA) was originally designed to obtain wide-field photographs of the retina in pediatric cases [17]. However, the modified optical technique can be used to assess the anterior chamber angle. Good agreement between EyeCam and conventional gonioscopy findings has been reported [18]. The major advantage of this technique is its ability to visualize the angle in its entirety, compared with UBM and AS-OCT that provide only cross-sectional views. The disadvantages include lack of quantitative analysis, expense, supine position for exam, longer image acquisition time, inability to perform indentation gonioscopy, and influence of fiberoptic light source on angle recess assessment.

### 2.5. Anterior Segment Optical Coherence Tomography 

AS-OCT uses the principle of low-coherence interferometry instead of ultrasound to produce high-resolution, cross-sectional images of the anterior segment of the eye [19, 20]. The technique measures the delay and intensity of the light reflected from the tissue structure being analyzed and compares it with the light reflected by a reference mirror. The combination of these two signals results in interference phenomenon. The signal intensity depends on the optical properties of the tissues, and the device uses these signals to construct a sagittal cross-section image of the structure being analyzed. OCT technology was initially used to produce images of the posterior segment of the eye by using a wavelength of 820 nm [21–23]. In 2001, the wavelength was altered to 1310 nm to allow better penetration through light-retaining tissues such as the sclera and limbus and to improve visualization of the anterior segment [24, 25]. 

Compared with UBM, this technology provides a higher axial resolution (18 um versus 25 um in 50 MHz UBM) and faster sampling rate (2.0 kHz versus 0.8 kHz). Another main clinical advantage over UBM is its ability to provide noncontact scanning in a seated, upright position. However, the image acquisition can be affected at times by the superior eyelid, and oblique angles may allow cross-sectional images. In addition, image distortions may result from off-axis measurements, requiring special software correction to eliminate the influence of scanning angle and refractive index of the cornea [26]. Lack of a coupling medium may affect the image quality due to abnormalities in the anterior surface of the eye [27, 28]. The major drawback for AS-OCT is its inability to visualize structures posterior to the iris due to blockage of wavelength by pigment [29, 30]. This limits its application in discerning several secondary causes of angle closure, such as plateau iris, ciliary body cyst or tumor, lens subluxation, or ciliary effusions.

The two AS-OCT devices commercially available are Visante-OCT (Carl Zeiss Meditec; CA, USA) and slit-lamp OCT (SL-OCT; Heidelberg Engineering GmbH, Heidelberg, Germany). Compared with the Visante-OCT, the SL-OCT has lower axial and transverse resolution, slower image acquisition, and requires manual rotation of the scanning beam. The properties of Visante-OCT are listed in Table 1. Leung et al. [31] reported high interobserver reproducibility with Visante-OCT and SL-OCT but poor agreement between the two devices. The authors speculated that differences in instrumentation, scan speed, and scan resolution may be responsible for observed differences. In another study, both devices detected more closed angles than did conventional gonioscopy [32]. However, better agreement was noted between SL-OCT and gonioscopy, presumably because of the use of visible light during both procedures.

### 2.6. Normal Angle

The anterior chamber angle refers to the junction between the iris root and cornea. In evaluating the angle, an important anatomic landmark is the scleral spur, which is the connecting point between the posterior curvature of the cornea and the curvature of the sclera. Trabecular meshwork and Schwalbe's line are located anterior to the scleral spur. The iris root and the ciliary body are located posterior to the scleral spur. Once the scleral spur is identified, attention is paid to the position of the iris relative to the scleral spur. If the iris is posterior to the scleral spur, the angle is open. If the iris is anterior to the scleral spur, the angle is either narrow or closed. 

### 2.7. Angle Parameters Measured with UBM and AS-OCT

Anterior segment imaging plays an important role not only in identifying the angle structures qualitatively, but also in providing quantitative measurements by using special software to determine the extent of apposition in cases of angle closure. After the scleral spur is located, several parameters can be measured [33–35]. Anterior chamber depth is defined as the axial distance between the posterior surface of the cornea and anterior lens surface. One important parameter for angle anatomy is the angle opening distance (AOD): the length of line drawn perpendicular from a point on the corneal endothelial surface (either 500 or 750 um anterior to the scleral spur) to the iris surface [36]. Theoretically, a distance of 500 um from the scleral spur approximates the location of the trabecular meshwork, and a longer distance of 750 um, covering a more extensive region, may be less affected by local iris surface irregularities. The software also provides a linear regression of the AOD out to 750 um. A formula, *y* = *ax* + *b*, is calculated to measure acceleration (a) and the *y*-intercept (b) to describe various types of angle configurations. Acceleration indicates the rate at which the angle widens from the scleral spur, and the *y*-intercept describes the distance from the scleral spur to the iris. Negative values for coefficients a and b indicate shallow depth at the central and peripheral parts of the angle, respectively [26]. 

Because AOD measurements are made in the iris plane, they can be influenced by the presence of peripheral anterior synechiae or other irregularities of iris contour and curvature. To overcome these limitations and to account for the whole contour of the iris surface, Ishikawa et al. [37] devised the angle recess area (ARA), which borders the anterior iris surface, corneal endothelium, and AOD 500 or AOD 750. Therefore, the ARA is defined as the triangular area with boundaries including the angle recess (apex), iris surface and the inner corneoscleral wall (sides), and AOD (base). The anterior chamber angle is defined in degrees, in which the angle recess forms the apex and the two sides of the angle are formed by drawing the lines through the points defining the AOD 500.

**Figure 1:**
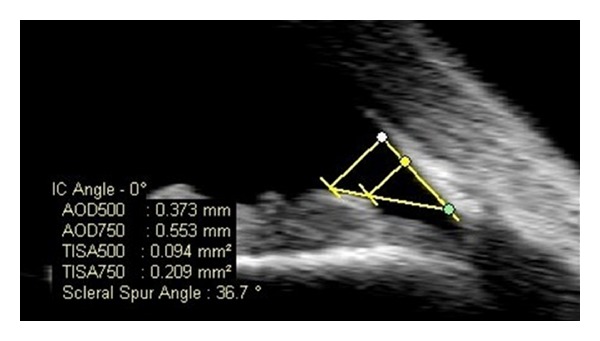
AS-OCT (anterior segment optical coherence tomography): quantitative measurements of angle parameters: green dot: scleral spur, connecting point between the posterior curvature of the cornea and the curvature of the sclera. yellow dot: linear distance of 500 um anterior to the scleral spur which marks the location of the trabecular meshwork, white dot: linear distance of 750 um anterior to the scleral spur which covers a more extensive area surrounding the trabecular meshwork. AOD 500 and AOD 750: Linear distance from the cornea to the iris at 500 and 750 um from the scleral spur, respectively. TISA 500 and TISA 750: Area of trapezoid between iris and cornea from sclera to 500 um and 750 um, respectively.

Because of poor visualization of the angle recess near the scleral spur and inability to measure the ARA properly with AS-OCT, Radhakrishnan et al. [25] proposed calculating the trabecular-iris space area (TISA), which does not require clear visualization of the angle recess (Figure 1). The researchers described this parameter to be a better indicator than ARA for actual filtering area and a more sensitive identifier of narrow angles in eyes with deep angle recesses. The TISA excludes the nonfiltering area via its posterior border outlined by a line drawn from the scleral spur to the opposing iris perpendicular to the plane of the inner scleral wall. Therefore, the TISA is the trapezoidal area with AOD anteriorly, inner scleral wall posteriorly, inner corneoscleral wall superiorly, and iris surface inferiorly. The same investigators also defined the trabecular-iris contact length (TICL) that can be used to denote an anatomically closed angle. The TICL is the linear distance of iris contact with corneoscleral surface beginning at the scleral spur and extending anteriorly. Therefore, this parameter measures the length of contact between iris and angle structures anterior to the scleral spur. 

Identifying the scleral spur is a critical landmark for both UBM and AS-OCT before calculating other angle parameters. Scleral spur location is reported to be successful in approximately 72% of images obtained with AS-OCT [6]. The difficulty in visualizing the scleral spur was mostly seen in areas where images were superior or inferior of the nasal and temporal quadrants. Good intraobserver reproducibility and poor interobserver reproducibility have been reported for these parameters with UBM, with high interobserver variation being attributed to manual identification of the scleral spur that could influence other angle parameters [38, 39]. However, Radhakrishnan et al. [25, 40] reported good intra- and interobserver reproducibility in the nasal and temporal quadrants but more variation in the inferior quadrants when using the prototype version of Visante-OCT. Nolan et al. [41] demonstrated better detection of closed angles with AS-OCT than with gonioscopy, particularly in the superior and inferior quadrants, although inadvertent pressure from gonioscopy lens or room illumination may have accounted for these findings. Li et al. [42] reported high intraobserver and interobserver reproducibility with AS-OCT in both light and dark conditions, but this study was limited by enrolling only healthy subjects and analyzing only the nasal angle.

## 3. Clinical Applications of AS-OCT

### 3.1. Closed-Angle Mechanisms

**Figure 2:**
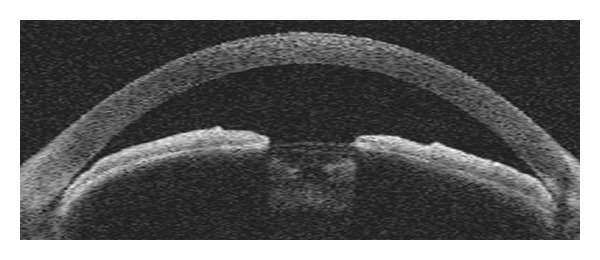
Qualitative assessment of narrow angle and shallow anterior chamber by AS-OCT.

**Figure 3:**
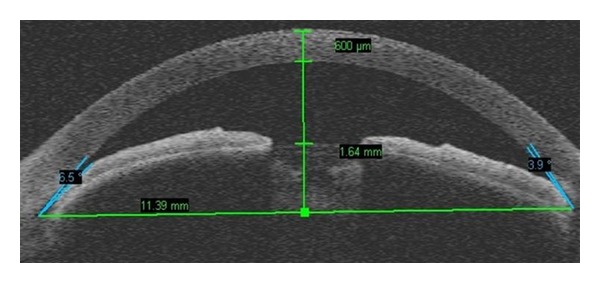
Quantitative assessment of narrow angle and shallow anterior chamber by AS-OCT.

Anatomically narrow angles can be diagnosed with AS-OCT both qualitatively and quantitatively (Figures 2 and 3). Radhakrishnan et al. [25] imaged 31 eyes, including both normal subjects and subjects with narrow angles, and compared results of UBM and the prototype version of AS-OCT by using the same customized software to conventional gonioscopy under similar room illumination. Values for AOD, ARA, TISA, and TICL were similar between UBM and AS-OCT. The same investigators also showed high specificity and sensitivity in detecting narrow angles with these two devices when compared with gonioscopy. Nolan et al. [41] reported high sensitivity, but low specificity of AS-OCT when compared with gonioscopy. Widening of the angles after laser iridotomy in eyes with narrow angles or pupillary block glaucoma has been demonstrated with both UBM and AS-OCT [43, 44].

**Figure 4:**
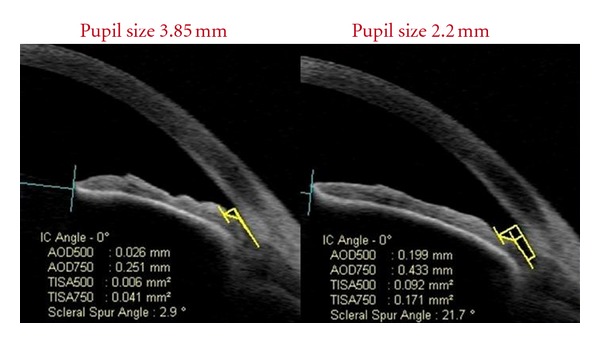
Influence of pupil size on angle measurements obtained with AS-OCT; the angle is more open (21.7° versus 2.9°) with a smaller pupil.

As different parameters are calculated to assess the angle, it is important to keep in mind that various factors, such as room illumination, accommodation, or medications may affect the shapes and locations of anterior segment structures (Figure 4). In addition, identifying scleral spur, the most important anatomic landmark for locating the trabecular meshwork (located 250–500 um anterior to the scleral spur), remains a subjective measure. Failure to properly identify the scleral spur can induce errors in subsequent measurements of angle parameters. Cheon et al. [45] studied the effect of age on anterior chamber angle parameters by AS-OCT. They reported lower values with negative slopes for AOD, TISA, ARA, and anterior chamber depth. Therefore, the influence of age should be considered when assessing changes in the anterior chamber over time with this technology.

**Figure 5:**
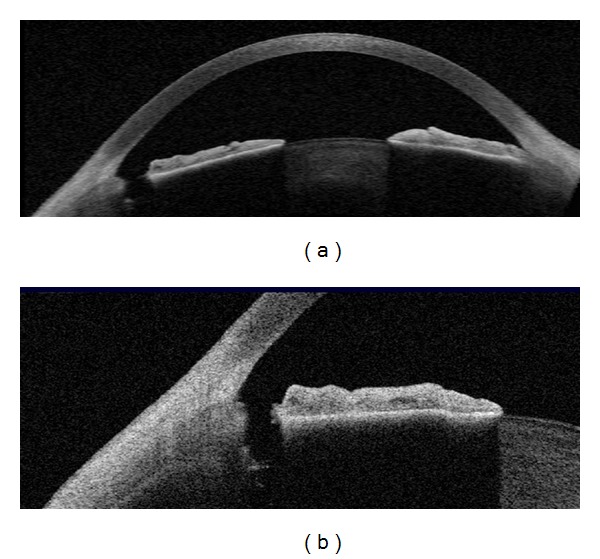
Patent peripheral iridotomy with AS-OCT. (a) shows enhanced anterior segment image with patent peripheral iridotomy and (b) shows raw mode and high-resolution image of cross-section through the anterior chamber with patent peripheral iridotomy.

Angle closure is characterized by apposition of the peripheral iris to the trabecular meshwork, resulting in obstruction of aqueous outflow. A variety of mechanisms involving the iris, ciliary body, lens, or forces posterior to the lens may be involved in the pathogenesis. Of various etiologies, pupillary block is the most common cause of angle closure and results from lens-iris contact, creating a pressure differential between the posterior and anterior chambers. The increased pressure in the posterior chamber leads to iris convexity with closure of the anterior chamber angle. Laser iridotomy is the definitive treatment for this condition. Equalizing the pressure differential in the two compartments reverses the iris bombe configuration and opens the drainage angle. AS-OCT can be used to diagnose this condition and monitor response to laser iridotomy (Figure 5).

**Figure 6:**
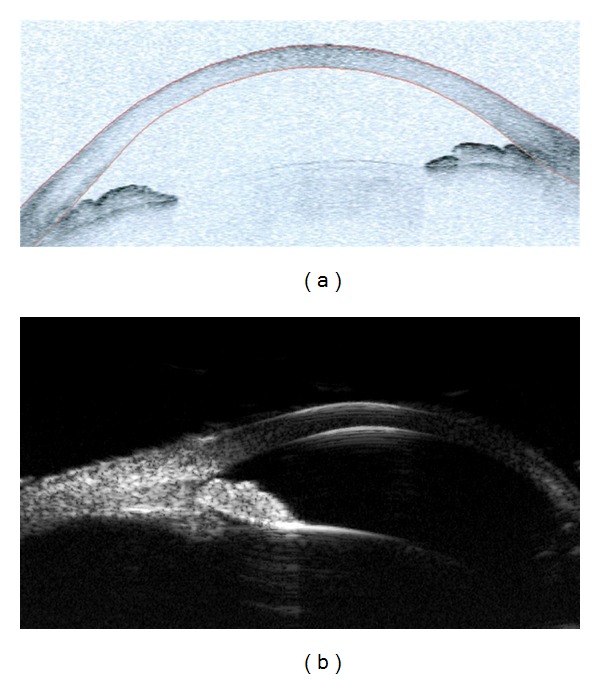
AS-OCT and UBM (ultrasound biomicroscopy) images in an eye with plateau iris syndrome. AS-OCT (a) shows closed angle in anterior segment single-mode image, but is limited in visualization of pathology posterior to the iris. UBM (b) shows anterior rotation of the ciliary body and assists in the diagnosis of plateau iris syndrome.

Although AS-OCT is limited due to blockage of infrared light by iris pigment with incomplete visualization of the ciliary body, it can visualize iris cyst, iris melanoma, or ciliary effusions in some cases. AS-OCT has been described to differentiate cystic and solid lesions of the iris [46]. However, these pathologies and others including plateau iris, phacomorphic glaucoma, or malignant glaucoma are better detected with UBM. In contrast to AS-OCT, UBM better demonstrates the anterior rotation of the ciliary body with loss of ciliary sulcus in plateau iris syndrome (Figure 6). 

### 3.2. Open-Angle Mechanisms

**Figure 7:**
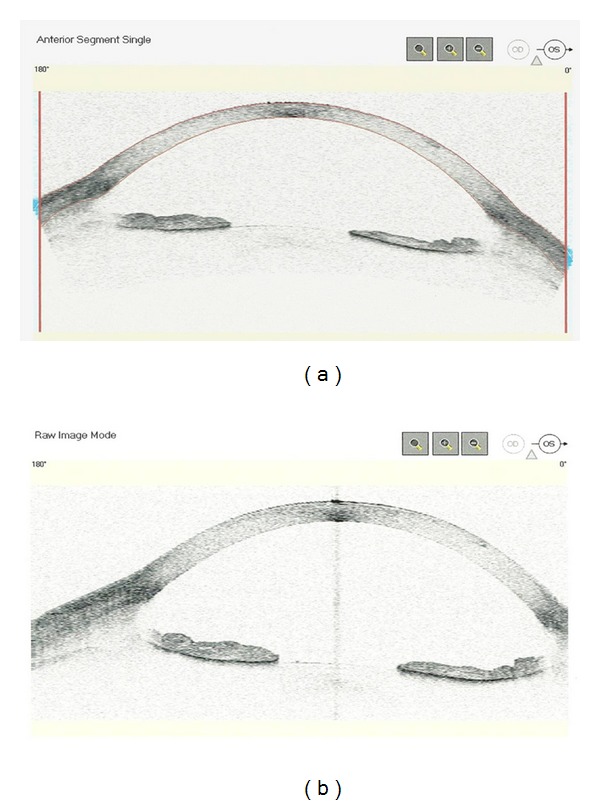
AS-OCT: Raw and anterior segment single mode images of preblinking (a) in an eye with pigment dispersion syndrome; postblinking image (b) shows increased iris concavity, deeper anterior chamber, and a wider angle from increased iridolenticular contact as a result of blinking.

AS-OCT can be used to assess the iris contour in pigment dispersion syndrome (PDS). In this condition, pigment liberation is secondary to rubbing between the iris pigment epithelium and lens zonules because of increased iridolenticular contact. The rubbing results from posterior bowing or concavity of the iris, which, in turn, is due to a reverse pressure gradient between the anterior and posterior chambers. In essence, there is a reverse pupillary block caused by blinking in which aqueous humor is pumped into the anterior chamber by movement of the iris but is prevented from flowing backward because of the valve effect of the iris against the lens (Figure 7). Aptel et al. [47] used AS-OCT to demonstrate that increased iridolenticular contact in PDS is not due to an abnormally large iris relative to the anterior segment size but to the reverse pressure gradient between the two chambers. The authors reported decreased anterior chamber volume and iridolenticular contact after laser iridotomy but increased iris volume-to-length ratio, suggesting higher deformability of the iris in PDS. While UBM and AS-OCT have shown benefit of laser peripheral iridotomy in eliminating the reverse pressure gradient and posterior bowing of the iris, whether these alterations in the contour of the iris favorably influence the long-term course of the intraocular pressure in these patients remains unknown.

**Figure 8:**
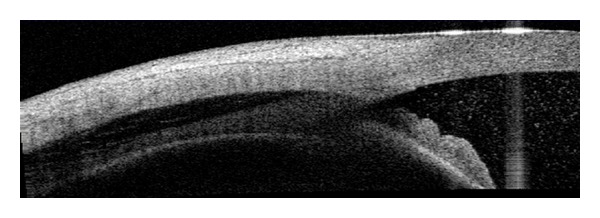
AS-OCT: trauma with a BB gun resulting in hyphema, angle recession, and choroidal hemorrhage.

The noncontact nature of AS-OCT makes it a valuable tool in identifying angle pathology in posttraumatic eyes (Figure 8). Angle recession [48] or cyclodialysis cleft may be documented.

### 3.3. Postsurgical Management

**Figure 9:**
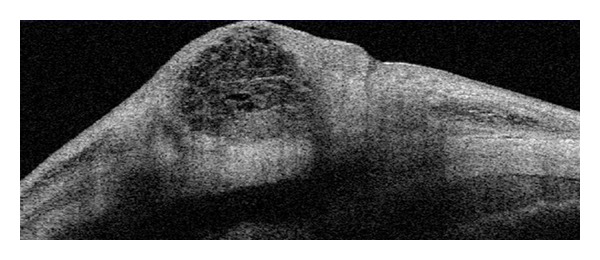
AS-OCT: functioning bleb after standard trabeculectomy.

**Figure 10:**
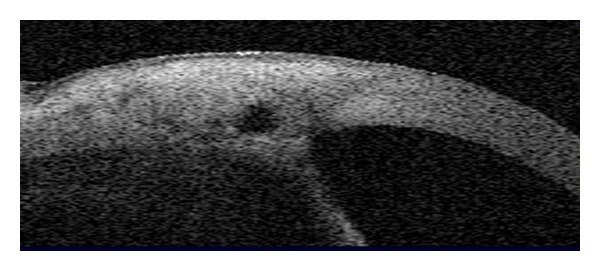
AS-OCT: non-functioning bleb after standard trabeculectomy.

**Figure 11:**
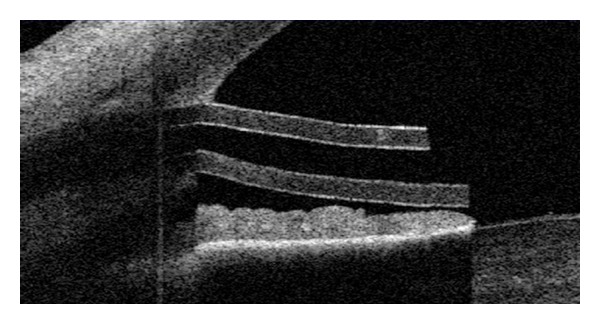
AS-OCT: patent Ahmed gaucoma valve.

AS-OCT is a useful tool to evaluate filtering blebs or glaucoma drainage devices in the postoperative period (Figures 9, 10, and 11). Clinically, blebs can be described as diffuse, cystic, encapsulated, or flat. However, these descriptions are subjective and there may be cases in which clinical appearance does not correlate with bleb function. Therefore, visualizing intrableb morphology with anterior segment imaging may enhance our understanding of different surgical outcomes and wound healing. Although several studies have described the UBM findings of filtering and nonfunctioning blebs, the noncontact AS-OCT scanning provides a significant advantage over UBM in eliminating direct trauma to the bleb or reducing the risk of potential infection that could occur with the use of an eye-cup or probe [36, 49–51]. In addition, the higher scanning resolution of AS-OCT allows differentiating the subconjunctival fluid collection and the suprascleral fluid space. Leung et al. [51] used AS-OCT to describe intrableb morphology and structures, including bleb wall thickness, subconjunctival fluid collections, suprascleral fluid space, scleral flap thickness, and intrableb intensity. They demonstrated low to medium intrableb reflectivity and intrableb fluid-filled spaces in functioning blebs (diffuse and cystic blebs). Encapsulated blebs had a thick wall, high reflectivity because of dense collagenous connective tissue present in the bleb wall, and an enclosed fluid-filled space. Flat blebs demonstrated high scleral reflectivity with no bleb elevation. Although qualitative assessment can be performed, the authors did caution that specific software does not exist for quantitative analysis of blebs and that the measurements in the study may not have reflected true values. However, the measurements could be used to compare different types of blebs and monitor bleb changes over time. Bleb morphology after nonpenetrating deep sclerectomy has also been reported [52]. AS-OCT allows visualization of the glaucoma drainage devices in the anterior chamber to assess their position or potential occlusion.

### 3.4. Measurement of Central Corneal Thickness (CCT)

**Figure 12:**
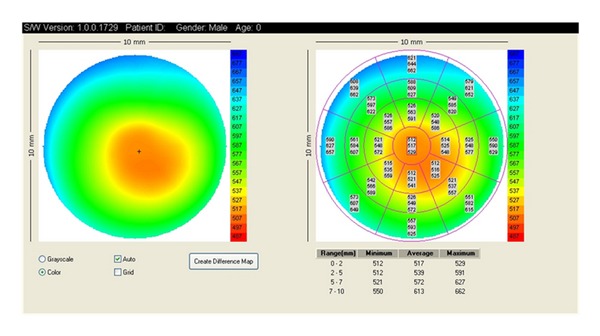
AS-OCT: pachymetry map.

It is well established that CCT influences the accuracy of intraocular pressure measurements obtained with the Goldmann applanation tonometer. Therefore, measuring CCT has become the routine component of glaucoma evaluation. Several types of pachymeters are available for measuring CCT, but ultrasound pachymetry is considered the standard because of its established reliability. However, this technique is limited by being a contact technique, and errors can be introduced by using a probe that can lead to misplacement or corneal compression. AS-OCT has built-in analysis software to measure the CCT automatically without contact with the eye (Figure 12). In addition, AS-OCT allows both central and regional pachymetry. Several studies reported thinner measurements of central cornea with retinal OCT than with ultrasound pachymetry [53, 54]. Using Visante-OCT, Li et al. [55] demonstrated a smaller difference in measurements with AS-OCT and ultrasound pachymetry by averaging the measurements over the central 2 mm rather than using a single focal measurement. Other investigators reported a reproducible systematic difference in CCT measurement obtained with SL-OCT and ultrasound pachymetry and Visante-OCT and ultrasound pachymetry, concluding that measurements obtained by AS-OCT and ultrasound pachymetry are not interchangeable [56–58]. In another study, Li et al. [58] evaluated the repeatability and reproducibility of central corneal thickness measurements obtained by SL-OCT and Visante-OCT and compared their agreement with ultrasound pachymetry. No significant difference was noted between automatic/manual SL-OCT and ultrasound pachymetry. The automatic Visante-OCT measured thinner than did ultrasound pachymetry (535.7 ± 30.2 um versus 550 ± 31.14 um; *P* < 0.001). In contrast, CCT measurement with manual Visante-OCT was higher than with ultrasound pachymetry (558 ± 32.8 um; *P* < 0.001). Nevertheless, both imaging devices had 95% limits of agreement with ultrasound pachymetry as demonstrated in the Bland-Altman plots. 

Clinically, it is important for clinicians to be aware of the differences in CCT measurements between AS-OCT and ultrasound pachymetry and caution should be exercised in interpreting CCT obtained from different anterior segment imaging systems. 

## 4. Conclusion 

While no technology can be a substitute for a thorough clinical examination performed by an experienced ophthalmologist, AS-OCT is a valuable adjunctive tool for anterior segment imaging, especially the angle anatomy in glaucoma suspects and patients. Its noncontact nature, high-resolution images, rapid scanning speed, storage capacity, imaging in the presence of corneal opacities, and the ability to provide both qualitative and quantitative analyses of the angle recess make it an important diagnostic tool for disease documentation, progression, and therapeutic outcomes. Its limitations should be kept in mind, including cost and its inability to image the ocular structures posterior to the iris due to blockage of wavelength by pigment.

## Figures and Tables

**Table 1:** The visante AS-OCT (anterior segment optical coherence tomography) properties.

Wavelength	1310 nm
Axial resolution	18 um
Transverse resolution	60 um
Depth of penetration	6 mm
Acquisition time	0.125 seconds per cross-section for overall anterior segment examination 0.25 seconds per cross-section for high resolution corneal examination
Image size	6 mm in depth by 16 mm wide for overall view of the anterior segment 3 mm in depth by 10 mm wide for high resolution Images
Coupling medium	Air
Patient position	Upright, seated
Operator requirement	Simple, non-contact test

## References

[1] Quigley HA (1996). Number of people with glaucoma worldwide. *British Journal of Ophthalmology*.

[2] Dandona L, Dandona R, Mandal P (2000). Angle-closure glaucoma in an urban population in Southern India. The Andhra Pradesh Eye Disease Study. *Ophthalmology*.

[3] Foster PJ, Oen FTS, Machin D (2000). The prevalence of glaucoma in chinese residents of singapore: a cross-sectional population survey of the tanjong pagar district. *Archives of Ophthalmology*.

[4] Foster PJ, Johnson GJ (2001). Glaucoma in china: how big is the problem?. *British Journal of Ophthalmology*.

[5] Ritch R, Liebmann J, Tello C (1995). A construct for understanding angle-closure glaucoma: the role of ultrasound biomicroscopy. *Ophthalmology Clinics of North America*.

[6] Sakata LM, Lavanya R, Friedman DS (2008). Comparison of gonioscopy and anterior segment ocular coherence tomography in detecting angle closure in different quadrants of the anterior chamber angle. *Ophthalmology*.

[7] Scheie HG (1957). Width and pigmentation of the angle of the anterior chamber; a system of grading by gonioscopy. *A.M.A. Archives of Ophthalmology*.

[8] Spaeth GL (1971). The normal development of the human anterior chamber angle: a new system of descriptive grading. *Transactions of the Ophthalmological Societies of the United Kingdom*.

[9] Foster PJ, Devereux JG, Alsbirk PH (2000). Detection of gonioscopically occludable angles and primary angle closure glaucoma by estimation of limbal chamber depth in Asians: modified grading scheme. *British Journal of Ophthalmology*.

[10] Aung T, Lim MCC, Chan YH, Rojanapongpun P, Chew PTK (2005). Configuration of the drainage angle, intraocular pressure, and optic disc cupping in subjects with chronic angle-closure glaucoma. *Ophthalmology*.

[11] Pavlin CJ, Harasiewicz K, Sherar MD, Foster FS (1991). Clinical use of ultrasound biomicroscopy. *Ophthalmology*.

[12] Barkana Y, Dorairaj SK, Gerber Y, Liebmann JM, Ritch R (2007). Agreement between gonioscopy and ultrasound biomicroscopy in detecting iridotrabecular apposition. *Archives of Ophthalmology*.

[13] Kaushik S, Jain R, Pandav S, Gupta A (2006). Evaluation of the anterior chamber angle in Asian Indian eyes by ultrasound biomicroscopy and gonioscopy. *Indian Journal of Ophthalmology*.

[14] Ritch R, Liebmann JM (1998). Role of ultrasound biomicroscopy in the differentiation of block glaucomas. *Current Opinion in Ophthalmology*.

[15] Hockwin O, Weigelin E, Laser H, Dragomirescu V (1983). Biometry of the anterior eye segment by Scheimpflug photography. *Ophthalmic Research*.

[16] Rabsilber TM, Khoramnia R, Auffarth GU (2006). Anterior chamber measurements using Pentacam rotating Scheimpflug camera. *Journal of Cataract and Refractive Surgery*.

[17] Erraguntla V, MacKeen LD, Atenafu E (2006). Assessment of change of optic nerve head cupping in pediatric glaucoma using the RetCam 120. *Journal of AAPOS*.

[18] Perera SA, Baskaran M, Friedman DS (2010). Use of eyecam for imaging the anterior chamber angle. *Investigative Ophthalmology and Visual Science*.

[19] Huang D, Swanson EA, Lin CP (1991). Optical coherence tomography. *Science*.

[20] Konstantopoulos A, Hossain P, Anderson DF (2007). Recent advances in ophthalmic anterior segment imaging: a new era for ophthalmic diagnosis?. *British Journal of Ophthalmology*.

[21] Sourdille P, Santiago PY (1999). Optical coherence tomography of macular thickness after cataract surgery. *Journal of Cataract and Refractive Surgery*.

[22] Hee MR, Puliafito CA, Duker JS (1998). Topography of diabetic macular edema with optical coherence tomography. *Ophthalmology*.

[23] Pieroth L, Schuman JS, Hertzmark E (1999). Evaluation of focal defects of the nerve fiber layer using optical coherence tomography. *Ophthalmology*.

[24] Radhakrishnan S, Rollins AM, Roth JE (2001). Real-time optical coherence tomography of the anterior segment at 1310 nm. *Archives of Ophthalmology*.

[25] Radhakrishnan S, Goldsmith J, Huang D (2005). Comparison of optical coherence tomography and ultrasound biomicroscopy for detection of narrow anterior chamber angles. *Archives of Ophthalmology*.

[26] Dorairaj S, Liebmann JM, Ritch R (2007). Quantitative evaluation of anterior segment parameters in the era of imaging. *Transactions of the American Ophthalmological Society*.

[27] Hoerauf H, Wirbelauer S, Scholz C (2000). Slit-lamp-adapted optical coherence tomography of the anterior segment. *Graefe’s Archive for Clinical and Experimental Ophthalmology*.

[28] See JLS, Chew PTK, Smith SD (2007). Changes in anterior segment morphology in response to illumination and after laser iridotomy in Asian eyes: an anterior segment OCT study. *British Journal of Ophthalmology*.

[29] van den Berg TJTP, Spekreijse H (1997). Near infrared light absorption in the human eye media. *Vision Research*.

[30] van den Berg TJTP, Spekreijse H (1997). Near infrared light absorption in the human eye media. *Vision Research*.

[31] Leung CKS, Li H, Weinreb RN (2008). Anterior chamber angle measurement with anterior segment optical coherence tomography: a comparison between slit lamp OCT and visante OCT. *Investigative Ophthalmology and Visual Science*.

[32] Sakata LM, Wong TTL, Wong HT (2010). Comparison of Visante and slit-lamp anterior segment optical coherence tomography in imaging the anterior chamber angle. *Eye*.

[33] Pavlin CJ, Foster FS (1995). Ultrasound biomicroscopic anatomy of the normal eye and adnexa. *Ultrasound Biomicroscopy of the Eye*.

[34] Marchini G, Pagliarusco A, Toscano A, Tosi R, Brunelli C, Bonomi L (1998). Ultrasound biomicroscopic and conventional ultrasonographic study of ocular dimensions in primary angle-closure glaucoma. *Ophthalmology*.

[35] Cho HJ, Woo JM, Yang KJ (2002). Ultrasound biomicroscopic dimensions of the anterior chamber in angle-closure glaucoma patients. *Korean Journal of Ophthalmology*.

[36] Pavlin CJ, Harasiewicz K, Foster FS (1992). Ultrasound biomicroscopy of anterior segment structures in normal and glaucomatous eyes. *American Journal of Ophthalmology*.

[37] Ishikawa H, Uji Y, Emil K (1995). A new method of quantifying angle measurements based on ultrasound biomicroscopy. *Atarashii Ganka*.

[38] Tello C, Liebmann J, Potash SD, Cohen H, Ritch R (1994). Measurement of ultrasound biomicroscopy images: intraobserver and interobserver reliability. *Investigative Ophthalmology and Visual Science*.

[39] Urbak SF, Pedersen JK, Thorsen TT (1998). Ultrasound biomicroscopy. II. Intraobserver and interobserver reproducibility of measurements. *Acta Ophthalmologica Scandinavica*.

[40] Radhakrishnan S, See J, Smith SD (2007). Reproducibility of anterior chamber angle measurements obtained with anterior segment optical coherence tomography. *Investigative Ophthalmology and Visual Science*.

[41] Nolan WP, See JL, Chew PTK (2007). Detection of primary angle closure using anterior segment optical coherence tomography in Asian eyes. *Ophthalmology*.

[42] Li H, Leung CKS, Cheung CYL (2007). Repeatability and reproducibility of anterior chamber angle measurement with anterior segment optical coherence tomography. *British Journal of Ophthalmology*.

[43] Gazzard G, Friedman DS, Devereux JG, Chew P, Seah SKL (2003). A prospective ultrasound biomicroscopy evaluation of changes in anterior segment morphology after laser iridotomy in Asian eyes. *Ophthalmology*.

[44] Chalita MR, Li Y, Smith S (2005). High-speed optical coherence tomography of laser iridotomy. *American Journal of Ophthalmology*.

[45] Cheon MH, Sung KR, Choi EH (2010). Effect of age on anterior chamber angle configuration in Asians determined by anterior segment optical coherence tomography; Clinic-based study. *Acta Ophthalmologica*.

[46] Siahmed K, Berges O, Desjardins L, Lumbroso L, Brasseur G (2004). Anterior segment tumor imaging: advantages of ultrasound (10, 20 and 50 MHz) and optical coherence tomography. *Journal Francais d’Ophtalmologie*.

[47] Aptel F, Beccat S, Fortoul V, Denis P (2011). Biometric analysis of pigment dispersion syndrome using anterior segment optical coherence tomography. *Ophthalmology*.

[48] Kawana K, Yasuno Y, Yatagai T, Oshika T (2007). High-speed, swept-source optical coherence tomography: a 3-dimensional view of anterior chamber angle recession. *Acta Ophthalmologica Scandinavica*.

[49] Yamamoto T, Sakuma T, Kitazawa Y (1995). An ultrasound biomicroscopic study of filtering blebs after mitomycin C trabeculectomy. *Ophthalmology*.

[50] Singh M, Chew PTK, Friedman DS (2007). Imaging of trabeculectomy blebs using anterior segment optical coherence tomography. *Ophthalmology*.

[51] Leung CKS, Yick DWF, Kwong YYY (2007). Analysis of bleb morphology after trabeculectomy with Visante anterior segment optical coherence tomography. *British Journal of Ophthalmology*.

[52] Nozaki M, Kimura H, Kojima M, Ogura Y (2002). Optical coherence tomographic findings of the anterior segment after nonpenetrating deep sclerectomy. *American Journal of Ophthalmology*.

[53] Bechmann M, Thiel MJ, Neubauer AS (2001). Central corneal thickness measurement with a retinal optical coherence tomography device versus standard ultrasonic pachymetry. *Cornea*.

[54] Wong ACM, Wong CC, Yuen NSY, Hui SP (2002). Correlational study of central corneal thickness measurements on Hong Kong Chinese using optical coherence tomography, Orbscan and ultrasound pachymetry. *Eye*.

[55] Li Y, Shekhar R, Huang D (2006). Corneal pachymetry mapping with high-speed optical coherence tomography. *Ophthalmology*.

[56] Zhao PS, Wong TY, Wong WL, Saw SM, Aung T (2007). Comparison of central corneal thickness measurements by visante anterior segment optical coherence tomography with ultrasound pachymetry. *American Journal of Ophthalmology*.

[57] Kim HY, Budenz DL, Lee PS, Feuer WJ, Barton K (2008). Comparison of central corneal thickness using anterior segment optical coherence tomography vs ultrasound pachymetry. *American Journal of Ophthalmology*.

[58] Li H, Leung CKS, Wong L (2008). Comparative study of central corneal thickness measurement with slit-lamp optical coherence tomography and visante optical coherence tomography. *Ophthalmology*.

